# Visualisation of peroxisomes: a journey through seven decades

**DOI:** 10.1007/s00418-026-02492-8

**Published:** 2026-05-20

**Authors:** Xinyu Tang, Markus Islinger, Michael Schrader

**Affiliations:** 1 Faculty of Health and Life Sciences, Department of Biosciences, University of Exeter, Exeter, EX4 4QD UK; 2 Institute of Neuroanatomy, Mannheim Center for Translational Neuroscience, Medical Faculty Mannheim, University of Heidelberg, 68167 Mannheim, Germany

**Keywords:** Peroxisomes, Mammalian, Yeast, Plant, Cytochemistry, Electron microscopy, Immunolabelling

## Abstract

Peroxisomes are ubiquitous, oxidative organelles in eukaryotes with conserved functions in cellular lipid metabolism and redox balance. They are now recognised as key metabolic organelles of medical importance, with contributions to antiviral signalling, pathogen defence, innate immunity, cancer and neurodegeneration. Our current knowledge of peroxisomes and the molecular understanding of their diverse functions and roles in disease have been achieved through a combination of biochemical and morphological studies. This review provides a comprehensive overview of the morphological techniques and approaches developed over the past 70 years to specifically label, identify and visualise peroxisomes in mammals, yeast and plants. We discuss the historical milestones that led to the first visualisation of peroxisomes in these organisms and trace the evolution of imaging strategies from early cytochemical and electron microscopy methods to modern fluorescence-based and high-resolution techniques. By highlighting the contribution of these approaches to advances in peroxisome biology, this review aims to place current peroxisome visualisation methods in a historical and methodological context.

## Introduction

Peroxisomes are single membrane-bound, oxidative organelles with a finely granular matrix. They are ubiquitously found in (almost all) eukaryotes and have fascinated scientists since their discovery 70 years ago (Rhodin 1954) owing to their highly plastic and dynamic nature, responsiveness to environmental changes, their interesting biology and importance for human health and disease. Peroxisomes are key metabolic organelles with essential functions in cellular lipid metabolism and the maintenance of cellular redox balance. They contain several flavin oxidases which consume oxygen and generate hydrogen peroxide, which in turn is decomposed by catalase, a prominent peroxisomal marker enzyme; hydrogen peroxide also acts as an important signalling molecule (Mittler et al. 2022; Fransen and Lismont 2024). Peroxisomes in mammals are involved in the alpha- and beta-oxidation of complex fatty acids (e.g. branched-chain fatty acids or very-long-chain fatty acids, which cannot be degraded by mitochondrial beta-oxidation), the synthesis of ether phospholipids (e.g. required in the myelin sheath), bile acids and several polyunsaturated fatty acids [such as docosahexaenoic acid (DHA), an important membrane component particularly in the retina and brain], as well as in purine, polyamine, glyoxylate and amino acid metabolism (Wanders and Waterham 2006). Peroxisomal functions across organisms, species and even tissues and cells can be very versatile (Islinger et al. 2010). In filamentous fungi, peroxisomes have key roles in the synthesis of antibiotics (e.g. penicillin) and other secondary metabolites (van der Klei and Veenhuis 2013). Plant peroxisomes harbour the glyoxylate cycle to convert fatty acids into carbohydrates (e.g. glyoxysomes in seeds and seedlings) and are involved in the biosynthesis of phytohormones (e.g. jasmonic acid), photorespiration and reactive oxygen species (ROS) metabolism (Hu et al. 2012; Pan et al. 2020; Corpas et al. 2020). In trypanosomatides, peroxisomes contain glycolytic enzymes, so are therefore called glycosomes (Andrade-Alviarez et al. 2022). Besides their metabolic functions, additional, often protective roles of peroxisomes in the combat of cellular stress, healthy ageing, pathogen defence and the regulation of immune responses have been discovered (Sandalio et al. 2021; Ferreira et al. 2022; Di Cara et al. 2023), which has moved peroxisomes centre stage in modern cell biology and biomedicine.

A characteristic feature of peroxisomes is their marked ability to proliferate, which is reflected by changes in number, size, shape and enzyme composition in response to metabolites (e.g. fatty acids) or a diverse group of chemical compounds (so-called peroxisome proliferators, such as hypolipidemic drugs and phthalate esters) (Hess et al. 1965; Reddy and Lalwai 1983). In yeasts and plants, environmental conditions are often driving peroxisome proliferation, e.g. methanol induction in the yeast *Hansenula polymorpha* (syn. *Ogataea polymorpha*), and salt stress or light in plants (see Sects. “Visualisation of peroxisomes in yeast cells” and “Visualisation of peroxisomes in plant cells”). Their biogenesis, which involves the insertion of membrane proteins, the import of proteins/enzymes in the peroxisomal matrix and their multiplication/proliferation, depends on several peroxins (PEX proteins), which represent biogenesis factors encoded by PEX genes (reviewed by Jansen et al. 2021; Kumar et al. 2024). They were initially identified through mutagenesis studies in yeast exploiting the proliferation of peroxisomes induced by carbon sources such as oleic acid. Peroxisomes can form out of pre-existing peroxisomes by a process of membrane growth and division (reviewed in Schrader et al. 2016; Carmichael and Schrader 2022) but can also form de novo under certain conditions (Hoepfner et al. 2005; Sugiura et al. 2017). They can import completely folded or even oligomeric proteins, likely through a pore-like protein complex, which is still under debate (Barros-Barbosa et al. 2019; Rudowitz and Erdmann 2023; Skowyra et al. 2024).

Defects in PEX proteins can result in severe peroxisome biogenesis disorders (PBDs) with developmental defects, organ abnormalities and neurological alterations, as observed in Zellweger spectrum disorders (ZSDs) (Steinberg et al. 2020; Wanders et al. 2023). Under those conditions, peroxisomes lose their metabolic functions as they can no longer import matrix proteins, and only empty membrane remnants (“ghosts”) can be detected, or peroxisomes can also be completely absent because of defects in membrane assembly. This results in an accumulation of toxic peroxisomal substrates (e.g. very-long-chain fatty acids) or a shortage of peroxisomal products (e.g. ether phospholipids or DHA). In addition, single enzyme deficiencies (SEDs) have been identified, which are based on defects in individual peroxisomal enzymes/proteins, affecting mainly one metabolic pathway of peroxisomes. These include deficiencies in peroxisomal beta-oxidation enzymes or defects in ABCD1, a peroxisomal ABC transporter for the uptake of very-long-chain fatty acids, causing X-linked adrenoleukodystrophy (Wanders et al. 2023; Engelen 2024). As a result of their contribution to the regulation of cellular redox balance, peroxisomes have also been linked to cellular ageing, age-related disorders, neurodegeneration and cancer (Fransen et al. 2013; Islinger et al. 2018; Pratama et al. 2024).

Our current knowledge about peroxisomes and the molecular understanding of their multiple functions and roles in disease could only be achieved by a combination of biochemical and morphological studies. Here, we provide an overview of morphological techniques and approaches, which have been developed in the last 70 years to specifically label, identify and visualise peroxisomes in mammals, yeast and plants. We will also address when and how peroxisomes were first visualised in those organisms. The visualisation and investigation of peroxisomes in other model organisms such as zebrafish and flies have recently been reviewed (Pridie et al. 2020; Jiang and Schrader 2025).

## Visualisation of peroxisomes in mammalian tissues and cells

### Early detection of mammalian peroxisomes by electron microscopy through their characteristic morphological features

In 2024, we celebrated the 70th anniversary of the discovery of peroxisomes (microbodies). The organelle was first described by the Swedish microscopist J. Rhodin in the proximal convoluted tubule cells of the mouse kidney (Rhodin 1954). Using transmission electron microscopy, he described a special type of cytoplasmic body, characterised by a single, limiting membrane and a finely granular matrix, which he named “microbody”. Soon after, “microbodies” were also described in the rat liver using electron microscopy (Bernhard and Rouiller 1956), containing a dense core with a regular crystalloid structure, which was later identified as urate oxidase (Hruban and Swift 1964). These morphological studies combined with biochemical studies by Christian De Duve and co-workers, who were the first to isolate peroxisomes and to characterise them biochemically (reviewed in De Duve 1996). Cell-fractionation experiments and determination of enzyme activity revealed that in rat liver, urate oxidase, d-amino acid oxidase, and catalase were associated together in a special group of cytoplasmic particles not identical to lysosomes (De Duve et al. 1960). Similar particles were identified in rat kidney and the ciliate protozoa *Tetrahymena pyriformis*, containing additional oxidases acting on glycolate, l-α hydroxyacids and l-amino acids (Wattiaux et al. 1964; Baudhuin et al. 1965b). The presence of hydrogen peroxide-producing flavin oxidases as well as catalase, which can decompose hydrogen peroxide, in those particles led De Duve to propose the functional name “peroxisome” for this new, apparently widely distributed cell organelle (De Duve 1965). Electron microscopy morphometric studies by Hess et al. (1965) revealed that hypolipidemic drugs can induce a marked proliferation of peroxisomes in hepatocytes of rodents (Reddy and Lalwai 1983).

**Fig. 1:**
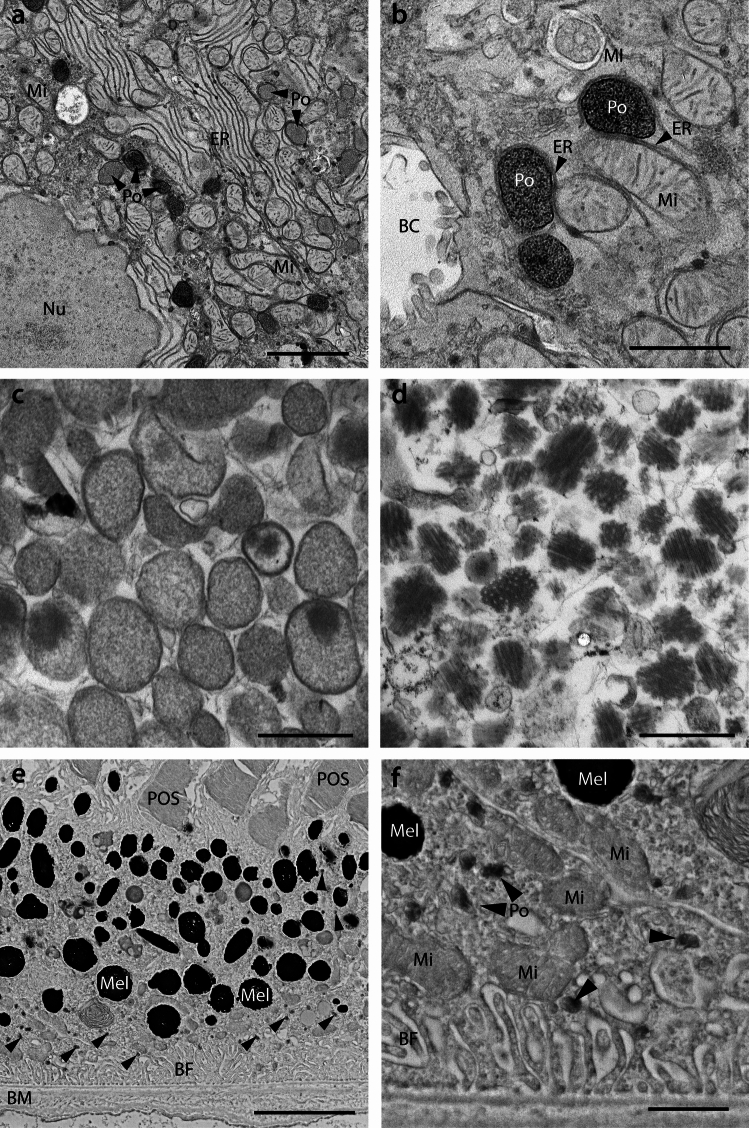
Ultrastructure of mammalian peroxisomes. **a** Peroxisomes in mouse liver hepatocytes were marked for d-amino acid oxidase activity using the cerium technique (Angermuller and Fahimi 1988; Islinger et al. 2010). Note that peroxisomes show heterogeneous staining indicating varying d-amino acid oxidase activities in neighbouring hepatocytes and even inside one cell. Scale bar 2 µm. **b** Peroxisomes in the same specimen as shown in **a** in a higher magnification view. As indicated by arrowheads, peroxisomes are frequently apposed to ER tubules, which often also maintain contacts with mitochondria in the direct vicinity. Scale bar 1 µm. **c** Liver peroxisomes isolated from a heavy mitochondrial fraction using free flow electrophoresis (Islinger et al. 2010). Peroxisomes show a homogenous granular appearance and occasionally contain a crystalline core of urate oxidase. Scale bar 0.5 µm. **d** Peroxisomal core fraction from the same experiment as shown in **c**. Note the regular crystalline structure cut in sagittal or horizontal planes. Scale bar 0.5 µm. **e** Microperoxisomes in the retinal pigment epithelium (RPE) of the mouse identified by alkaline DAB staining (Merz et al. 2025). Peroxisomes indicated by arrowheads are preferentially found in the basolateral region of the cells. Scale bar 5 µm. **f** Magnification from **e**. A comparison to neighbouring mitochondria highlights the significantly smaller size of RPE compared to hepatocyte peroxisomes. Scale bar 1 µm. *Po* peroxisomes, *Mi* mitochondria, *Nu* nucleus, *ER* endoplasmic reticulum, *BC* bile canaliculus, *Mel* melanosomes, *POS* photoreceptor outer segments, *BF* basal folds, *BM* Bruch’s membrane

The application of cell fractionation and density gradient centrifugation allowed researchers to enrich and isolate peroxisomes (microbodies) and to visualise the isolated organelles by electron microscopy, with isolated rat liver peroxisomes revealing a crystalline core of urate oxidase facilitating their identification (Baudhuin et al. 1965a; Leighton et al. 1968) (Fig. 1). Characteristic crystalline inclusions were also later observed in peroxisomes from other mammalian species, enabling their identification (Böck et al. 1980; Islinger et al. 2010). These included the marginal plates in peroxisomes of the bovine kidney cortex which consist of α-hydroxyacid oxidase B and are located directly below the peroxisomal membrane, giving the peroxisomes a characteristic angular shape (Zaar et al. 1986, 1991). A recent ultrastructural study also described marginal plates in peroxisomes in the kidney of the dromedary camel (Eissa et al. 2024).

Furthermore, the application of freeze-fracture and freeze-etching techniques contributed to the morphological characterisation of peroxisomes. The presence of crystalline inclusions in peroxisomes, e.g. in the rat or bovine kidney, supported their identification in freeze-etch preparations. The technique also allowed visualisation of membrane particles on the fracture faces of the peroxisomal membrane, as well as a close association of peroxisomes with the endoplasmic reticulum (ER) (Kalmbach and Fahimi 1978; Gorgas and Zaar 1984; Zaar and Fahimi 1990; Kryvi et al. 1990; Furuta et al. 1992). It also contributed, together with the DAB cytochemistry (see Sect. “Visualising mammalian peroxisomes by DAB cytochemistry for peroxisomal catalase: the development of a peroxisome-specific staining method”), to the identification of peroxisomes in trypanosomatids (Souto-Padrón and de Souza 1982).

### Visualising mammalian peroxisomes by DAB cytochemistry for peroxisomal catalase: the development of a peroxisome-specific staining method

The first specific staining method for peroxisomes was based on the peroxidase substrate 3,3′-diaminobenzidine (DAB), which was initially introduced by Graham and Karnovsky (1966) for the localization of horseradish peroxidase by electron microscopy. H. D. Fahimi then revealed that DAB can specifically stain peroxisomes, which is dependent on the peroxidatic activity of peroxisomal catalase (Fahimi 1968, 1969, 2009; Hirai 1968; Novikoff and Goldfischer 1969). Subsequently, the conditions for the alkaline DAB method for the staining of peroxisomal catalase were optimised, revealing that aldehyde fixation increased the peroxidatic activity of catalase. Furthermore, DAB incubation at high-alkaline pH (10.5), elevated temperature and a high concentration (0.15%) of hydrogen peroxide improved DAB intensity and specificity of the staining (reviewed in Fahimi and Baumgart 1999; Deimann et al. 1991; Fahimi 2017). The oxidised DAB forms an electron-dense, insoluble polymer within the peroxisomal matrix, which can be visualised by electron microscopy, but also by light microscopy as a brownish-dark precipitate, e.g. in histological sections (Fig. 1e). The electron density of DAB can be further enhanced by post-fixation with osmium tetroxide, resulting in the formation of osmium black.

The availability of a peroxisome-specific staining method supported the characterisation of peroxisomes in different species, tissues and cell types, and greatly contributed to the recognition of peroxisomes as ubiquitous organelles of eukaryotic cells (Hruban et al. 1972). The application of the DAB technique also revealed the absence of peroxisomes in the liver and kidney of patients with Zellweger syndrome, thus leading to the identification of the first peroxisomal disorder in humans (Goldfischer et al. 1973). Subsequent studies applied DAB cytochemistry to examine biopsies from patients with suspected peroxisomal disorders (Roels et al. 1987) or in knockout mouse models with defects in peroxisome biogenesis (Baes et al. 1997). Moreover, DAB staining has been used to quantify changes in peroxisome number and shape/size induced by peroxisome proliferators/xenobiotics using electron microscopy morphometry and light microscopy automated image analysis (Beier and Fahimi 1986, 1987). The DAB method has been adopted for the staining of peroxisomes in cultured cells (Stier et al. 1998; Grabenbauer et al. 2000; Bonekamp et al. 2013) and of isolated peroxisomes obtained by gradient centrifugation (Volkl and Fahimi 1985).

### Mapping oxidative activity: visualising peroxisomal oxidase activity in mammals by the cerium technique

Light microscopy techniques for the localisation of peroxisomal oxidases were introduced for the detection of urate oxidase in the liver (Graham and Karnovsky 1965) and the localisation of α-hydroxyacid oxidase activity in the kidney (Allen and Beard 1965), but those methods were not applicable for electron microscopy. Cerium was introduced by Briggs et al. (1975) for the detection of hydrogen peroxide generated by NADH-oxidase in granulocytes. The method was then modified for the localisation of peroxisomal oxidases in the methanol-grown yeast *Hansenula polymorpha* (Veenhuis et al. 1976) (see Sect. “Cerium cytochemistry and yeast peroxisomes: towards an understanding of peroxisome architecture”). As a result of the sensitivity of peroxisomal oxidases to fixation, optimised protocols for vertebrate tissues had to be developed (Angermuller and Fahimi 1986). Incubation in cerium chloride with specific oxidase substrates (e.g. urate, glycolate or d-proline) results in the formation of electron-dense cerium perhydroxide as the final reaction product of cerium chloride and hydrogen peroxide (Fig. 1a, b). Sodium azide had to be added to inhibit catalase which would otherwise decompose the hydrogen peroxide generated by the oxidases. Furthermore, appropriate buffers such as PIPES or Tris–HCl buffer had to be used (Angermuller 1989). As cerium perhydroxide lacks sufficient contrast in light microscopy, DAB–nickel or cobalt chloride was introduced, which improved visualisation of the cerium reaction product by transmitted light (Angermuller and Fahimi 1988).

The cerium method allowed one to localise oxidase activity directly within peroxisomes. It confirmed that key H_2_O_2_-producing enzymes are concentrated in these organelles, strengthening the idea that peroxisomes are specialised oxidative compartments. The visualisation of oxidase activity in combination with immune electron microscopy (see Sect. “Visualisation of mammalian peroxisomes by immunolabelling techniques: defining organelle identity and function”) has revealed that peroxisomes are heterogeneous organelles. Peroxisome enzymatic heterogeneity has been observed in the same tissue such as in liver lobules, and even within the same cell as shown in rat liver and kidney (Yamamoto and Fahimi 1987; Angermuller and Fahimi 1988), or in isolated subpopulations of peroxisomes (Luers et al. 1993). A heterogeneous oxidase/enzyme composition as observed in liver lobules may be explained by different oxygen concentrations in the periportal and pericentral regions. Furthermore, morphological heterogeneity of peroxisomes was observed including small and large spherical shapes as well as elongated peroxisomes, and membrane protrusions or “tails” (Gorgas 1984, 1987; Yamamoto and Fahimi 1987). The different peroxisomal morphologies as well as their close association with the ER (Fig. 1b) have inspired hypotheses and investigations about the biogenesis of peroxisomes. Peroxisomal membrane elongation (growth) and subsequent constriction and division of those tubules has been shown to contribute to peroxisome multiplication by growth and division of pre-existing peroxisomes (Lazarow and Fujiki 1985; Schrader et al. 1996; Schrader and Fahimi 2006). These processes depend on the peroxisomal membrane shaping protein PEX11β, and the membrane adaptors FIS1 and MFF, which can recruit the large dynamin-related fission GTPase DRP1 to the peroxisomal membrane (reviewed in Schrader et al. 2016; Carmichael and Schrader 2022). Remarkably, FIS1, MFF and DRP1 are also involved in mitochondrial division. Meanwhile, it has also been revealed that the peroxisomal membrane protein ACBD5 interacts with ER-resident VAP proteins to tether peroxisomes to the ER (Costello et al. 2017; Hua et al. 2017). Close contacts of peroxisomes with the ER have been observed on isolated peroxisomes in early ultrastructural studies (Zaar et al. 1987). The peroxisome–ER membrane contacts are involved in phospholipid transfer from the ER to peroxisomes, which is required for peroxisomal membrane expansion/elongation (Costello et al. 2017), and dysfunctions in peroxisome membrane dynamics have been linked to human disorders (Carmichael et al. 2022). Furthermore, the ER has been implicated in the de novo formation of peroxisomes in yeast (see Sect. “Visualisation of peroxisomes in yeast cells”) and mammalian cells under conditions where peroxisomes are absent (Hoepfner et al. 2005; Sugiura et al. 2017). These and other studies (e.g. Luers et al. 1993; Titorenko et al. 2000) have led to the identification of pre-mature and mature peroxisome populations implicated in the biogenesis of peroxisomes.

It was also first concluded from studies using cerium cytochemistry, together with immunoelectron microscopy, that peroxisomal enzymes/oxidases form distinct sub-compartments within the peroxisomal matrix. For example, in addition to the crystalline urate oxidase core and the marginal plates underneath the peroxisomal membrane, non-crystalline, focal condensations composed of d-amino acid oxidase in the centre of the matrix have been observed. A compartmentalisation of the peroxisomal matrix and membrane has been confirmed by recent super-resolution microscopy studies (Galiani et al. 2016; Ast et al. 2022; reviewed in Galiani et al. 2023) (see Sect. “Visualising peroxisomes beyond the diffraction limit: advances from super-resolution microscopy”). Furthermore, molecular insight into protein condensation has been revealed in studies using the filamentous fungus *Ustilago maydis* (Backer et al. 2025). The authors identified enzymes from the fungus, which accumulate inside of peroxisomal subdomains and describe a short peptide motif (Thr-Ile-Ile-Val), which is sufficient to trigger focal localisation, thus shedding light on the rather elusive mechanism of intra-peroxisomal compartmentalisation. Crystalline cores are also present in Woronin bodies, which are peroxisome-derived organelles in filamentous fungi that seal septal pores after damage, preventing cytoplasmic loss. Their core is formed by the protein Hex1, which assembles into a dense crystalline structure. This Hex1 crystal is essential for both the formation of Woronin bodies and their ability to rapidly plug pores and protect the cell (Jedd and Chua 2000).

### Visualisation of mammalian peroxisomes by immunolabelling techniques: defining organelle identity and function

The biochemical characterisation of peroxisomes revealed key marker proteins such as catalase and specific oxidases (see Sect. “Early detection of mammalian peroxisomes by electron microscopy through their characteristic morphological features”). Further biochemical and genetic characterisation disclosed additional functions of peroxisomes, e.g. in the beta-oxidation of fatty acids (Lazarow and De Duve 1976) and identified PEX proteins involved in peroxisome biogenesis, as well as peroxisomal membrane transporters (e.g. ABCD1–3 for fatty acid import). This enabled the generation of antibodies against peroxisomal matrix and membrane proteins, initially through isolation of the proteins and immunisation of rabbits. Evolving techniques for the expression and isolation of recombinant proteins in bacteria or insect cells, the use of peptides for antibody production, as well as methods to generate monoclonal antibodies or nanobodies (Muyldermans 2013) increased the antibody toolbox for peroxisomes. In addition to the localisation of endogenous peroxisomal proteins, candidate proteins were also cloned as fusions with different protein tags (e.g. MYC, FLAG), expressed in mammalian cells, and then localised based on antibodies directed against the protein tag. Those developments went hand-in-hand with the improvement of protocols for the fixation and processing of tissues and cells for immunolocalisation (reviewed in Usuda et al. 1995; Fahimi et al. 1996; Fahimi and Baumgart 1999; Baumgart et al. 2003). This also includes improvements in cryo-electron microscopy in combination with immunolabelling of peroxisomal proteins (e.g. Mildner and Zeuschner 2017).

**Fig. 2:**
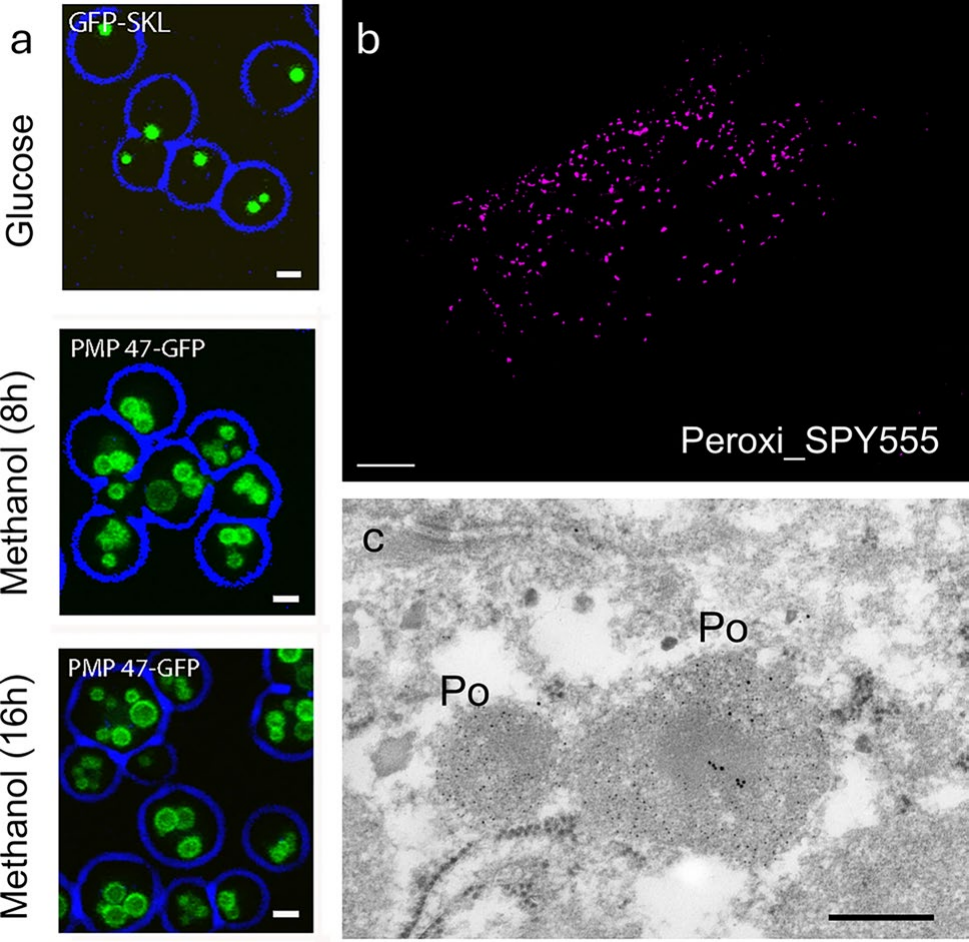
**a** Peroxisome proliferation in yeast. Fluorescence microscopy images of *Hansenula polymorpha* cells grown on glucose or methanol (8 h, 16 h), using confocal laser scanning microscopy. GFP-SKL was used to mark peroxisomes in glucose-grown cells, whereas PMP47-GFP was used to mark peroxisomes in methanol-grown cells. Scale bar 1 μm [adapted from Thomas et al. 2015. This figure is reproduced under the terms of the Creative Commons Attribution 4.0 International License (CC BY 4.0)]. **b** In vivo labelling of peroxisomes with Peroxi_SPY555 (Spirochrome). Human skin fibroblasts were incubated with Peroxi_SPY555 and imaged live using a motorised inverted fluorescence microscope (IX83; Olympus). A still image is shown. Scale bar 10 µm (kindly provided by M. Schuster and X. Tang, University of Exeter, UK). **c** Immunogold labelling of peroxisomes (PO) in rat liver. Samples were fixed with paraformaldehyde and glutaraldehyde (without OsO_4_), embedded in Epon, and post-embedding incubated with rabbit anti-catalase (5 nm gold particles) and guinea pig anti-‘copper chaperone of SOD1’ (CCS) antibodies (10 nm gold particles) and subsequent gold-labelled secondary antibodies. Scale bar 300 nm (for further information on CSS, see Islinger et al. 2009)

Initially, antibodies to peroxisomal catalase or the peroxisomal oxidases (e.g. urate oxidase, α-hydroxyacid oxidase) were used in immunoelectron microscopy to confirm the composition of the crystalline inclusions within peroxisomes and to investigate peroxisomal compartmentalisation (see Sect. “Mapping oxidative activity: visualising peroxisomal oxidase activity in mammals by the cerium technique”) (Fig. 2c). Subsequently, antibodies against the peroxisomal beta-oxidation enzymes (e.g. acyl-CoA oxidase, thiolase) and against membrane proteins (e.g. ABCD3/PMP70, PEX14, ACBD5) became available, although suitable antibodies for the morphological detection of peroxisomal membrane proteins and peroxins are still scarce.

The first immunocytochemical studies used ferritin- and peroxidase-labelled antibodies to visualise peroxisomal proteins in tissues (Yokota and Nagata 1974; Yokota and Fahimi 1981, 1982). Later on, the immunogold technique (Faulk and Taylor 1971; Roth et al. 1978) became the preferred method (e.g. Fahimi et al. 1996; Usuda et al. 1996), and protocols for the immunolabeling of peroxisomes in rodents and humans were developed (e.g. Litwin et al. 1984, 1987). Immunogold labelling and automated image analysis allowed the quantitative assessment of alterations in peroxisomal proteins after treatment of rodents and cell cultures with peroxisome proliferators (Reddy and Lalwai 1983; Beier et al. 1988; Ogiwara et al. 1999). Antibodies generated against the peroxisomal targeting signal of firefly luciferase supported the detection of other peroxisomal proteins in mammals carrying a PTS1 (Gould et al. 1990). Immunolabelling techniques have also greatly contributed to the investigation of peroxisomal disorders and are still valuable tools in their diagnosis (Roels et al. 1991; Wanders et al. 1995; Ferdinandusse et al. 2016). They revealed the heterogeneity of complementation groups in patients with Zellweger syndrome (Santos et al. 1992; Shimozawa et al. 1993). Furthermore, immunocytochemical and immunofluorescence studies contributed to the identification and characterisation of peroxisomal “ghosts”, which represent import-incompetent peroxisomal structures in peroxisome biogenesis disorders, which, however, still contain membrane proteins (Santos et al. 1988; Wiemer et al. 1989; Espeel et al. 1995). The visualisation of catalase by immunofluorescence in fibroblasts from patients with peroxisomal diseases supports the diagnosis of peroxisomal disorders (Wanders et al. 1989; Ferdinandusse et al. 2016). In controls, a punctate, peroxisomal staining is observed, whereas defects in several PEX proteins required for the biogenesis of peroxisomes impact peroxisomal protein import resulting in a cytosolic staining for catalase. Expression of individual PEX proteins in those patient cells can be used to identify which PEX gene is affected, as expression of the respective functional PEX protein will restore catalase import. Furthermore, aberrant peroxisome morphologies can be detected, which can be based on dysfunctional peroxisome division (e.g. based on defects in PEX11β, MFF or DRP1) (Waterham et al. 2007; Ribeiro et al. 2012; Carmichael et al. 2022). In those cases, the biochemical properties of the peroxisomes are normal or only slightly affected, and patients may not be identified based solely on peroxisomal biomarkers in plasma or blood.

Overall, immunolabelling techniques are widely used to e.g. investigate peroxisome biogenesis, protein import, peroxisomal disease, characterisation of peroxisomal knock out models, as well as for the verification of proteomics data identifying potentially new peroxisomal proteins (e.g. Islinger et al. 2007; Wiese et al. 2007; Gronemeyer et al. 2013; Yifrach et al. 2018; Singin et al. 2024). They contributed substantially to the recognition of the heterogeneity of peroxisomal protein composition in the different cell types of complex tissues (e.g. Ahlemeyer et al. 2007; Grant et al. 2013).

### Visualisation of mammalian peroxisomes by expression of fluorescent fusion proteins with a peroxisomal targeting signal: from static images to dynamic organelles

The visualisation of peroxisomes in living cells became possible with the discovery of the green fluorescent protein (GFP) from the jellyfish *Aequorea victoria* and its application in cell biology as a genetically encodable fluorescent tag (Chalfie et al. 1994; Rodriguez et al. 2017). This coincided with the discovery of PTS1, a major targeting signal for the sorting of proteins into the peroxisomal matrix, which consists of a C-terminal, tripeptide sequence (S/A/C-K/R/H-L/M) (Gould et al. 1989; Keller et al. 1991). A second targeting signal, PTS2, was found at the N-terminus of a much smaller subset of peroxisomal proteins in mammals (Osumi et al. 1991; Swinkels et al. 1991). Although the tripeptide SKL (serine-lysine-leucine) is sufficient to target GFP and other proteins to peroxisomes in many species, it has subsequently been revealed that the last C-terminal 12 amino acids of PTS1-containing proteins are important for targeting (Brocard and Hartig 2006).

In initial studies, peroxisomes in living mammalian cells were labelled by microinjection of FITC–luciferase to study peroxisome movement (Rapp et al. 1996). Firefly luciferase had been previously shown to target peroxisomes and was used to identify the PTS1 (Keller et al. 1987; Gould et al. 1989). Subsequently, Wiemer et al. (1997) generated a GFP-PTS1 fusion protein, which, when expressed in transfected mammalian cells, specifically targeted peroxisomes. They applied GFP-PTS1 to study peroxisome dynamics in living cells during interphase and mitosis using time-lapse confocal laser scanning microscopy. These and other studies provided first insight into the motile behaviour of mammalian peroxisomes and revealed that peroxisomes in mammalian cells move along microtubules, with only a small population exhibiting saltatory movements (Schrader et al 1996; Huber et al. 1997; reviewed in Schrader et al. 2003; Neuhaus et al. 2016). The discovery of peroxisome–ER contact sites in mammalian cells revealed that 60–80% of peroxisomes are in close contact with the ER, now explaining why a large peroxisome population is stationary. Accordingly, loss of the peroxisome–ER tether proteins ACBD5 or VAP results in increased mobility of peroxisomes visualised by GFP-PTS1 (Costello et al. 2017; Hua et al. 2017). Furthermore, binding and movement of isolated peroxisomes along microtubules in vitro could be visualised by video-enhanced differential interference contrast microscopy (Schrader et al. 2000).

The use of GFP-PTS1 also revealed the dynamic behaviour of complex peroxisomal structures in mammalian cells, such as tubular, elongated or reticular peroxisomes, and their association with lipid droplets or peroxisome–peroxisome interactions (Schrader et al. 2000; Schrader 2001; Bonekamp et al. 2012). Furthermore, a role for MIRO1, a membrane adaptor protein for kinesin and dynein motors, in peroxisome dynamics and motility was revealed (Okumoto et al. 2018; Castro et al. 2018). A GFP with a peroxisomal membrane anchor allowed MIRO1-dependent formation of peroxisomal protrusions from peroxisomal ghosts to be visualised in PEX5-deficient patient cells (Castro et al. 2018). These membrane protrusions, which have also been observed in yeast and plant cells (see Sects. “Visualisation of peroxisomes in yeast cells” and “Fluorescent fusion proteins and bioreporters in plant peroxisome research: new insights into peroxisome dynamics and physicochemical properties”) are thought to support organelle interplay and communication (reviewed in Carmichael et al. 2023).

It should be noted that peroxisome movement in yeast and plants is mediated by the actin cytoskeleton (see Sects. “Visualisation of peroxisomes in yeast cells” and “Visualisation of peroxisomes in plant cells”). However, filamentous fungi use microtubules for organelle movement, including peroxisomes. Interestingly, in those fungi peroxisomes do not directly interact with motor proteins; they instead move by hitchhiking on motile early endosomes, which has been revealed by live cell imaging of organelle-specific fluorescent reporters (Guimaraes et al. 2015; Salogiannis et al. 2016; Lin et al. 2016).

Meanwhile, sophisticated optogenetic tools have been developed, which allow for the optical control of intracellular transport and organelle positioning by using light-sensitive heterodimerization to recruit motor proteins to selected cargoes, including peroxisomes (van Bergeijk et al. 2015). Moreover, tools for the automated image analysis of peroxisome motility are available (e.g. Metz et al. 2017; Svenson et al. 2023), and mathematic modelling has been applied to understand their positioning and dynamics in cells (Lin et al. 2016; Castro et al. 2018; Passmore et al. 2020; Nicolaou et al. 2025). Recently, “perox-per-cell”, a software that processes fluorescence microscopy images to quantify peroxisome features such as counts per cell and spatial areas in yeast cells, has been introduced (Neal et al. 2024).

GFP-SKL and related fluorescent PTS1 fusion proteins have now developed into versatile reporters for the peroxisomal compartment, with many applications for the investigation of peroxisomal protein import, biogenesis, dynamics and peroxisome–organelle interplay, as well as for screening approaches and the determination of peroxisomal properties. A versatile toolbox of fluorescent and photoactive proteins has been used to visualise peroxisomal redox properties, hydrogen peroxide production, pH or pexophagy by fluorescence ratio measurements (Lismont et al. 2017; Godinho and Schrader 2017; Fransen and Lismont 2019; Costa et al. 2023; Barone et al. 2024); KillerRed has been applied to induce oxidative stress in peroxisomes and other cellular locations (Fransen and Brees 2017); split-GFP/SPLICS reporters have been used to determine peroxisome–organelle proximity or protein topology (Bishop et al. 2019; Vallese et al. 2020; Chornyi et al. 2024); HaloTag technology has been applied to fluorescently pulse-label peroxisomes allowing to optically distinguish pools of peroxisomal proteins that are synthesized at different time points (Huybrechts et al. 2009; Delille et al. 2010; Fransen 2014); HaloFlippers have been designed to measure membrane tension changes in living cells/organelles (Strakova et al. 2020). Moreover, near-infrared fluorescence and photoacoustic imaging have been applied to visualise peroxisomal viscosity (Zhou et al. 2020). Fluorescent peroxisomal reporter proteins were also used for correlative light and electron microscopy (CLEM) in peroxisome research. CLEM makes use of the advantages of protein localisation by fluorescence microscopy and combines them with the high resolution of electron microscopy. CLEM studies have contributed to the visualisation of peroxisome–organelle contacts in mammals and yeast (Ilacqua et al. 2022; de Boer and Van der Klei 2023), to peroxisomal protein localisation (Exner et al. 2019) and to the development of a high-throughput ultrastructure screening pipeline (Bykov et al. 2019). Furthermore, live-cell confocal and lattice light sheet spectral imaging approaches have been designed to map organelle numbers, volumes, speeds, positions and dynamic inter-organelle contacts in living mammalian cells for six different organelles (ER, Golgi, lysosomes, peroxisomes, mitochondria and lipid droplets) providing new insights into cellular organisation and dynamics (Valm et al. 2017).

To investigate protein–protein interaction of peroxisomal proteins or molecules, fluorescent proteins (or antibodies) have been used for fluorescence resonance energy transfer (FRET) measurements. Studies using FRET have shown that peroxisomal beta-oxidation enzymes form larger complexes (Wouters et al. 1998), characterised the interaction between PEX proteins, peroxisomal ABCD transporters, and quantified the interaction strength of PTS signals and their receptors in living cells (Muntau et al. 2003; Hillebrand et al. 2007; Krause et al. 2013; Hochreiter et al. 2020). Furthermore, FRET sensors have been used to visualise the peroxisomal redox state (Yano et al. 2010) and peroxisomal calcium dynamics (Drago et al. 2008; Sargsyan and Thoms 2023).

### Beyond genetic labelling: live cell imaging probes for mammalian/animal peroxisomes

Whereas specific live cell imaging probes for organelles such as lipid droplets (e.g. Nile Red, BODIPY 493/503), mitochondria (e.g. MITO-Tracker) or the nucleus/nuclear DNA (e.g. DAPI) are readily available, similar probes for live cell imaging of peroxisomes were lacking for a long time. An advantage of such probes is that they usually label organelles in minutes, whereas expression of fluorescent fusion constructs (e.g. GFP-PTS1) takes several hours and requires cell transfection. The first probe for mammalian peroxisomes was a peptide-based reporter, which consisted of a membrane-permeable PTS1 peptide carrying a fluorophore (e.g. fluorescein, BODIPY, SNAFL-2). When added to cultured mammalian cells, the fluorescent peptides were rapidly internalised and targeted to peroxisomes (Dansen et al. 2000; Pap et al. 2001) allowing investigation of morphology, dynamics and functional integrity of the peroxisomal matrix protein import machinery. Targeting of the pH-sensitive fluorophore SNAFL-2 to peroxisomes also revealed that peroxisomes in human fibroblasts and baker’s yeast have a basic pH (Dansen et al. 2000; van Roermund et al. 2004). Targeting of the PTS1 peptide reporters to peroxisomes was, however, dependent on an intact peroxisomal import machinery. Recently, fluorescent fatty acid conjugates for live cell imaging of peroxisomes were reported (Korotkova et al. 2024). These included BODIPY-C12 (which also labelled lipid droplets) and the novel, bright PeroxiSPY650 and PeroxiSPY555 probes, which contain longer fatty acids and were specific to peroxisomes (Fig. 2b). The probes stained functional and dysfunctional peroxisomes in live mammalian cells and were also applied to visualise peroxisomes in live zebrafish embryos. Fatty acids are imported into peroxisomes by the peroxisomal ATP-dependent transporters ABCD1–3, and it is suggested that the probes undergo a substrate-like import via those transporters. However, the probes did not stain peroxisomes in the plant *Arabidopsis thaliana*. Interestingly, a BODIPY-based probe for the in vivo labelling of peroxisomes in plants had been reported earlier (Landrum et al. 2010; Fahy et al. 2017) (see Sect. “Visualisation of peroxisomes in plant cells”), but it did not label peroxisomes in mammalian cells, highlighting differences between mammalian and plant peroxisomes, e.g. in substrate specificity and membrane composition.

### Visualising peroxisomes beyond the diffraction limit: advances from super-resolution microscopy

**Fig. 3:**
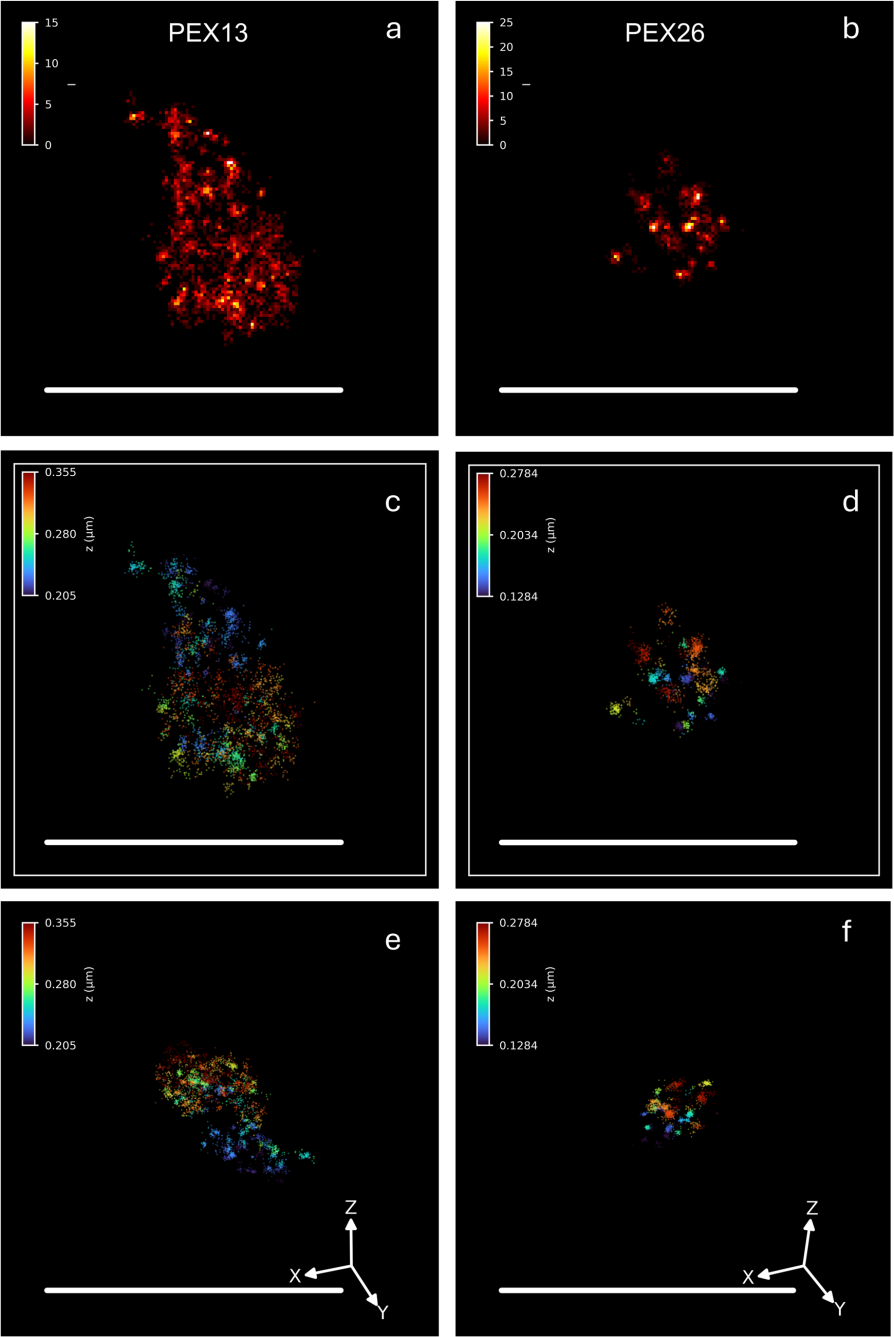
Super-resolution fluorescence microscopy (MINFLUX microscopy) of peroxisomal membrane protein distribution in mammalian cells. HEK293 cells were transfected with EGFP-PEX13 (**a**, **c**, **e**) or EGFP-PEX26 (**b**, **d**, **f**) and labelled with GFP-nanobodies coupled to Alexa-Fluor 647. A single peroxisome is shown. **a**, **b** Two-dimensional projection of a 3D MINFLUX recording. The colour code indicates the number of localisations (counts). **c**, **d** Z-colour-coded 3D recordings. **e**, **f** 3D presentation, rotated. The colour code indicates the Z-position. Scale bars 500 nm (kindly provided by J. Alvelid and K. Reglinski, University of Jena, DE)

The visualisation of peroxisomes by super-resolution microscopy (SRM) techniques has been recently reviewed (Galiani et al. 2023; Kumar et al. 2024), and we will therefore only briefly summarise major findings (see also Sect. “Advancing plant peroxisome research through super-resolution microscopy”). One of the first SRM images of peroxisomes was captured by single-molecule localisation microscopy (SMLM) after immunostaining of the peroxisomal ABC transporter ABCD3/PMP70 in fixed mammalian cells (Folling et al. 2008). Labelling of peroxisomes has often been used to show methodological improvements of SMLM, and protocols for the imaging of peroxisomes have been published (e.g. Halpern et al. 2015; Büttner et al. 2021; de Lange and Vlijm 2023; Klein et al. 2023; reviewed in Galiani et al. 2023). SRM has contributed to our understanding of the compartmentalisation of the peroxisomal matrix and membrane (Galiani et al. 2016; Ast et al. 2022) (see Sect. “Mapping oxidative activity: visualising peroxisomal oxidase activity in mammals by the cerium technique”) (Fig. 3), the morphological characterisation of ghost peroxisomes from patients with Zellweger spectrum disorders (Soliman et al. 2018), peroxisome biogenesis in the ischemic brain (Young et al. 2015), the diffusion and interaction dynamics of the import receptor PEX5 in the cytosol (Galiani et al. 2022), and the remodelling of peroxisome–ER contacts during viral infection (Cook et al. 2022). In the yeast *Hansenula polymorpha*, SRM has recently unveiled dynamic changes of Pex3 and the pexophagy receptor Atg30 in their localisation (de Lange et al. 2024).

## Visualisation of peroxisomes in yeast cells

### Introduction to yeast peroxisomes

**Fig. 4:**
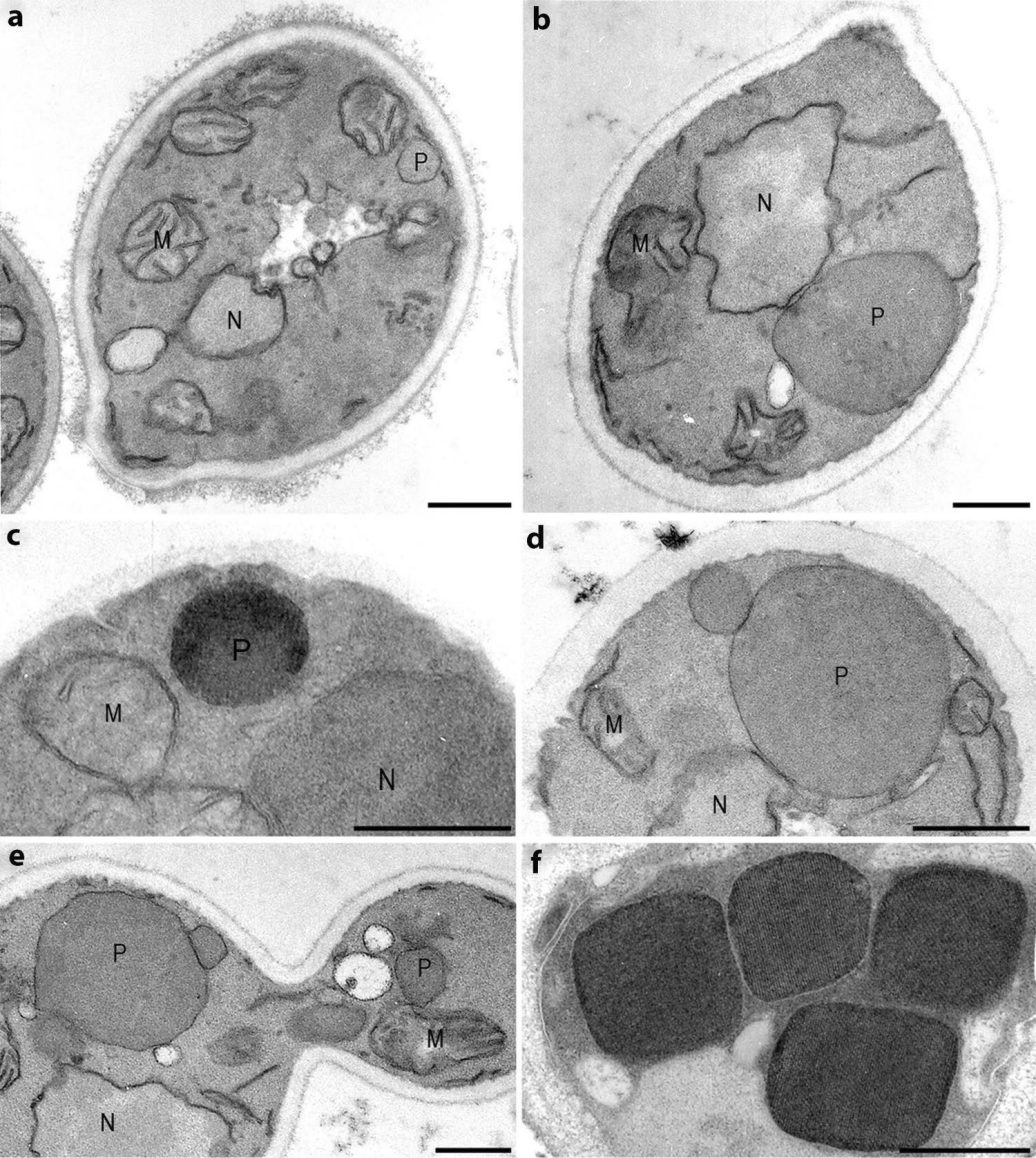
Imaging of peroxisome multiplication in the methylotrophic yeast *Hansenula polymorpha*. Cells are transferred from glucose- to methanol-containing medium, a condition in which peroxisomal activity becomes essential for growth. Under glucose conditions, cells typically contain a single peroxisome (**a**). Following the shift to methanol, this organelle accumulates key enzymes, including alcohol oxidase (**c**), catalase and dihydroxyacetone synthase, all required for methanol metabolism. Consequently, the peroxisome enlarges (**b**) and, upon maturation, undergoes fission to generate a daughter organelle (**d**), which then continues to grow. A comparable asymmetric division mechanism ensures proper inheritance of smaller peroxisomes to the budding daughter cell (**e**). In the late exponential growth phase, cells generally harbour 4–6 peroxisomes with similar morphology. Their characteristic cuboid appearance reflects the presence of large intraluminal alcohol oxidase crystals. *M* mitochondria, *N* nucleus, *P* peroxisome. Cells were fixed using KMnO_4_, except for **c**, **f**, which were treated with glutaraldehyde. In these samples, alcohol oxidase activity was detected using the CeCl_3_ technique. Scale bars 200 nm [from Veenhuis and Van der Klei 2014. This figure is reproduced under the terms of the Creative Commons Attribution 4.0 International License (CC BY 4.0)]

Yeast peroxisomes are essential metabolic organelles involved in fatty acid beta-oxidation, the glyoxylate cycle and methanol metabolism, as well as contributing to biosynthetic processess such as lipid and amino acid metabolism (Sibirny 2016). Unlike in animals, yeast cells only contain a peroxisomal and not a mitochondrial beta-oxidation pathway for fatty acids (see Sect. “Introduction”). Yeast peroxisomes also exhibit remarkable plasticity, with their size and number strongly regulated by the type of carbon source available. In distinct yeast species, several inducers such as methanol, long-chain fatty acids/oleate, methylamine or urate, d-amino acids and alkanes effectively stimulate peroxisome proliferation, whereas repressive carbon sources such as glucose and ethanol trigger the degradation of existing peroxisomes (Veenhuis et al. 1987; Sibirny 2016) (Figs. 2a, 4). As a result of their genetic tractability and metabolic relevance, yeast species such as *Saccharomyces cerevisiae*, *Hansenula polymorpha* (syn. *Ogataea polymorpha*) or *Pichia pastoris* (syn. *Komagataella phaffii*) have a long-standing history as excellent model organisms for studying the function, biogenesis and maintenance of peroxisomes. One major advantage of using yeast in peroxisome research is that defects or deficiencies in peroxisome function are non-lethal, e.g. when grown on glucose. This characteristic has made it possible to isolate mutants with impaired peroxisome biogenesis and to identify the corresponding genes through functional complementation (van der Klei and Veenhuis 2006). Most known PEX genes were discovered using such yeast mutants, and to date, 39 PEX genes have been identified as specifically involved in peroxisome biogenesis (Yuan et al. 2016; Sibirny 2016; Kumar et al. 2024). In addition, studies in yeast have been instrumental in our understanding of de novo peroxisome formation, peroxisome inheritance and peroxisome–organelle membrane contacts.

### DAB cytochemistry and the discovery of inducible peroxisomes in yeast cells

Yeast peroxisomes were first identified by Avers and Federman using electron microscopy in cells of *Saccharomyces cerevisiae* grown on glucose (Avers and Federman 1968). To investigate their enzymatic content and structure, DAB staining has been widely employed as a cytochemical method to localise catalase activity, a hallmark enzyme of peroxisomes (see also Sect. “Visualising mammalian peroxisomes by DAB cytochemistry for peroxisomal catalase: the development of a peroxisome-specific staining method”). Early cytochemical studies successfully visualised DAB oxidation products within peroxisomes in *S. cerevisiae* cells. Subsequent inhibition assays confirmed that DAB deposition in peroxisomes was specifically mediated by catalase activity, validating the specificity and reliability of this method for peroxisome identification (Hoffmann et al. 1970). The thick, glucan- and mannan-rich yeast cell wall restricts DAB penetration, resulting in faint peroxisome labelling. For example, conventional frozen sections showed only weak DAB staining in peroxisomes. However, the introduction of ultrathin frozen sectioning effectively resolved this limitation, significantly enhancing peroxisome visualisation without the need for cell wall digestion and allowing rapid sample preparation without embedding (Osumi and Sato 1978).

DAB staining also permitted tracking yeast peroxisome dynamics under different physiological conditions, e.g. monitoring changes in peroxisome number and morphology in response to shifts in nutrient conditions (e.g. carbon source changes). Notably, studies have demonstrated that catalase activity was significantly elevated in hydrocarbon- and methanol-grown yeasts compared to cells grown on glucose or ethanol (Osumi et al. 1974; Teranishi et al. 1974; Fukui et al. 1975). A representative example is observed in methanol-utilising yeasts, where peroxisomes (microbodies) enlarge and catalase activity increases following the transfer from malt extract medium to an *n*-alkane medium (Osumi et al. 1975). Furthermore, DAB staining revealed distinct patterns of catalase localisation: in methanol-grown cells, the reaction product was strongly concentrated within the crystalloids of the microbodies, whereas in hydrocarbon-grown cells, it was distributed more homogeneously throughout the microbody matrix (Fukui et al. 1975). In addition, DAB staining contributed to elucidating structural and functional defects in various peroxisomal mutants, e.g. the complete absence of recognisable peroxisomal structures in peroxisomal assembly-deficient mutants (Erdmann et al. 1989; Hohfeld et al. 1991) and aberrant peroxisomal clustering with impaired matrix protein import in identified peroxisome biogenesis mutants (Zhang et al. 1993). The induction of peroxisome proliferation by oleic acid was not reported until 1987 (Veenhuis et al. 1987). Consequently, early studies on yeast peroxisomes were performed with other yeast species, as outlined above. The discovery that growth on oleic acid both requires and induces peroxisomes in *S. cerevisiae* made it possible to exploit the extensive genetic tools available for this organism, which were not yet accessible for other yeasts. As a result, the first pex mutants were isolated in *S. cerevisiae*, and the first PEX gene (*PEX1*) was identified in this species.

However, DAB cytochemistry provided limited spatial resolution at the ultrastructural level and was insufficient for accurately delineating the fine architecture of the peroxisomal membrane and the topographical distribution of membrane-associated proteins in yeast. The combination of freeze fracture technology with DAB staining provided a powerful alternative. By exposing membrane surfaces through cryo-fracturing while preserving the native membrane organization, and visualising enzyme activity via DAB precipitation, this integrated approach significantly enhanced both the precision of protein localisation and the quality of structural imaging (Zwart et al. 1980; Veenhuis et al. 1983). To facilitate the recognition of peroxisomes, their proliferation was induced by altering the nutritional conditions (van Dijken et al. 1975; Tsubouchi et al. 1976), similar to approaches in rat liver, where peroxisomes were induced by hypolipidemic drugs (see Sect. “Early detection of mammalian peroxisomes by electron microscopy through their characteristic morphological features”).

### Cerium cytochemistry and yeast peroxisomes: towards an understanding of peroxisome architecture

As previously noted (see Sect. “Mapping oxidative activity: visualising peroxisomal oxidase activity in mammals by the cerium technique”), the cerium-based method was adapted for the localisation of peroxisomal oxidases in methanol-grown *H. polymorpha* (Veenhuis et al. 1976, 1992) (Fig. 4). Application of CeC_3_ cytochemistry provided critical insights into the heterogeneity of peroxisomes (Figs. 1 and 4). In yeast cells grown on glucose with methylamine as the sole nitrogen source, CeCl_3_ cytochemistry localised amine oxidase activity to specialised peroxisomes, demonstrating functional adaptation to nitrogen metabolism (Zwart et al. 1980). Similarly, in *H. polymorpha* grown on methanol, CeCl_3_ staining revealed highly ordered crystalline peroxisomal cores composed of active alcohol oxidase, illustrating structural specialisation for methanol metabolism (Veenhuis et al. 1981). In contrast, urate-grown yeasts such as *Candida famata* exhibited non-crystalline, homogeneous peroxisomes enriched in urate oxidase, highlighting substrate-specific architectural differences (Veenhuis et al. 1985). Further evidence for functional heterogeneity among microbodies in yeasts was provided by Veenhuis and colleagues, who demonstrated distinct enzyme compartmentalization and morphological differentiation among peroxisomal populations, thereby substantiating the concept of microbody specialisation under diverse physiological conditions (Veenhuis et al. 1989).

### Immunolabelling of yeast peroxisomes: implications for protein import and function

Immunogold labelling has proven to be a powerful technique for the subcellular localisation of peroxisomal proteins (see also Sect. “Visualisation of mammalian peroxisomes by immunolabelling techniques: defining organelle identity and function”). In yeast, it has been widely used to identify matrix enzymes such as alcohol oxidase, as well as membrane proteins (Douma et al. 1990; Van der Klei et al. 1991; Sulter et al. 1993). The technique has also been applied to identify the subcellular distribution of peroxisomal enzymes involved in beta-oxidation (Erdmann and Kunau 1994). A comprehensive review on immunogold labelling techniques in yeast has highlighted its effectiveness in studying protein targeting and morphological change (Binder et al. 1996). The application of immunogold labelling has enabled the differentiation of import-defective and peroxisome-deficient mutants, supporting the characterisation of specific PEX genes in peroxisomal biogenesis (Tan et al. 1995). This approach has also contributed to the understanding of the import mechanisms of peroxisomal matrix proteins via the PTS1 and PTS2 pathways (Rehling et al. 1996). Immunogold labelling has proven highly effective in revealing aberrant organelle morphology in yeast mutants defective in peroxisome formation or protein import. For instance, strains lacking Pex3p or Pex19p exhibit severe defects in peroxisome assembly (Hettema et al. 2000), while Pex1p-deficient mutants retain empty peroxisomal membrane structures, often referred to as “ghosts”, which lack matrix proteins (Knoops et al. 2015). Similarly, altered peroxisome number and size has been clearly visualised in other peroxisome-deficient mutants by immunogold labelling (Titorenko et al. 1995; Smith et al. 2000).

Later studies utilized antibodies raised against key peroxisomal matrix enzymes such as catalase and 3-ketoacyl-CoA thiolase to visualise their specific localisation within the peroxisomal matrix compartment (Thieringer et al. 1991; Zhang et al. 1993). Immunofluorescence using antibodies against the yeast carboxyl-terminal peroxisomal targeting signal (PTS) enabled specific visualisation and localisation of peroxisomal proteins across various yeast species (Aitchison et al. 1992). In addition to matrix proteins, antibodies directed against various peroxisomal integral membrane proteins, including peroxins (PEX proteins), have been applied to investigate the structural and regulatory roles of these components (Vizeacoumar et al. 2003).

To determine subcellular localisation, candidate proteins were cloned with epitope tags such as MYC or FLAG, expressed in yeast, and analysed using tag-specific antibodies alongside the detection of endogenous peroxisomal proteins. This approach was used to identify Pex13p as a peroxisomal membrane receptor essential for docking the PTS1 recognition factor Pex5p, thus playing a key role in matrix protein import (Erdmann and Blobel 1996). Separately, studies on Pex11p revealed that it promotes peroxisome division independently of metabolic function, highlighting its role in regulating organelle morphology rather than metabolism. These findings together advanced our understanding of peroxisome biogenesis by linking protein import machinery with organelle dynamics (Li and Gould 2002).

### Visualising yeast peroxisomes by fluorescent fusion proteins: insights into peroxisome dynamics, inheritance, de novo formation and organelle contacts

Monosov et al. demonstrated that GFP tagged with a C-terminal SKL sequence (GFP-SKL) effectively targets peroxisomes in living yeast cells (Monosov et al. 1996) (Fig. 2a). Building on this approach, subsequent studies utilized GFP (or DsRed)-SKL to reveal that peroxisome abundance and dynamics in *S. cerevisiae* or the fission yeast *Schizosaccharomyces pombe* are regulated by dynamin-related membrane fission proteins, specifically Vps1p and Dnm1p, and that the ordered segregation of peroxisomes into daughter cells is dependent on actin filaments (Faber et al. 2002; Kuravi et al. 2006). It was revealed that the actin cytoskeleton functions as tracks for the myosin motor Myo2p, driving directional peroxisome transport (Hoepfner et al. 2001). Furthermore, Myo2p interacts with peroxisomes through the adaptor protein Inp2p, ensuring proper distribution of peroxisomes into the daughter cell (Fagarasanu et al. 2005, 2006, 2009). Such targeted inheritance is critical for preventing the loss of functional peroxisomes in daughter cells and for preserving cellular metabolic capacity after division (Knoblach and Rachubinski 2016).

Additionally, GFP-PTS1 (or similar constructs) functioned as a live-cell fluorescent marker for peroxisomes, facilitating high-throughput and quantitative imaging of peroxisomal dynamics. This analytical strategy enabled the systematic screening of yeast libraries with thousands of yeast deletion mutants, leading to the identification of both established and previously uncharacterised factors involved in peroxisome biogenesis and PTS1-dependent matrix protein import and the discovery of new peroxisome functions (Wolinski et al. 2009; Yofe et al. 2017; Weill et al. 2018; Schummer et al. 2020; Yifrach et al. 2022). Moreover, an in-cell competition assay using increasing amounts of mCherry-PTS1 revealed targeting priority to yeast peroxisomes (Rosenthal et al. 2020).

Beyond peroxisomal targeting, GFP fusion to Pex proteins enabled researchers to study peroxisome biogenesis and proliferation. For instance, GFP-tagged peroxisomal proteins such as Pex11-GFP have illustrated the role of Pex11p in peroxisome proliferation and membrane elongation (Tam et al. 2003). GFP-Pex3p served as a marker for the peroxisome maturation pathway and played a crucial role in tracking peroxisome biogenesis. Using temperature-sensitive mutants of the methylotrophic yeast *H. polymorpha* and a combination of electron microscopy and immunocytochemistry, it was already suggested that peroxisomes do not necessarily derive from pre-existing organelles under certain conditions (Waterham et al. 1993). Reintroduction of GFP-Pex3p in *S. cerevisiae* Pex3 deletion mutants, which lack mature peroxisomes, revealed for the first time that peroxisomes can form de novo from the ER (Hoepfner et al. 2005), but continue to multiply by growth and division out of pre-existing organelles once formed (Motley and Hettema 2007). Subsequently, de novo formation of peroxisomes was also observed in other organisms and cell types, e.g. in mammalian cells under similar experimental conditions. The exact mechanism and physiological importance of de novo formation is still debated (Veenhuis and Van der Klei 2014; Yuan et al. 2016; Agrawal and Subramani 2016; Schrader and Pellegrini 2017; Wroblewska and Van der Klei 2019; Banerjee and Prinz 2023).

Moreover, fluorescent fusion proteins also facilitated the visualisation of peroxisome–organelle contact sites. For example, the use of Inp1p-GFP and Pex3p-mCherry contributed to their identification as a potential peroxisome–ER tether controlling peroxisome populations in *S. cerevisiae* (Knoblach et al. 2013). However, later studies showed that Inp1p functions as a peroxisome–plasma membrane tether (Hulmes et al. 2020; Krikken et al. 2020). Other studies have employed GFP-Pex3p to identify peroxisome–vacuole contact sites, revealing new aspects of peroxisome–organelle interactions and their spatial organization within the cell (Wu et al. 2019). A bimolecular fluorescence complementation (BiFC) analysis system was developed for the in vivo detection of protein–protein interactions in *S. cerevisiae* (Sung and Huh 2007). This technique enabled the direct visualisation of interactions in living yeast cells by reconstituting fluorescence when two proteins bring together split fragments of a fluorescent protein, allowing dynamic analysis of protein complexes. BIFC systems such as “split Venus” were applied in high-throughput screens to systematically analyse membrane contact sites. This led to the discovery of peroxisome–mitochondria tethers and a function for this membrane contact in the beta-oxidation of fatty acids in yeast (Shai et al. 2018) as well as of three previously unrecognized homologues of the lipid transporters Vps13, Atg2 and Csf1, all localised at multiple contact sites (Castro et al. 2022).

Beyond morphological analyses, the combination of live cell imaging with advanced quantitative proteomics, such as dual-track SILAC, has enabled real-time mapping of protein interaction networks within their native cellular context. This approach has revealed both stable and transient interactions of integral peroxisomal membrane proteins such as Pex30p, providing deeper insights into the molecular machinery that governs peroxisome dynamics (David et al. 2013).

## Visualisation of peroxisomes in plant cells

### Introduction to plant peroxisomes

Compared to animals, peroxisomes in plants play more complex roles in responding to environmental stresses and regulating plant-specific metabolic processes. These diverse functions are crucial for plant growth and development that involve processes such as lipid catabolism, photorespiration, ROS metabolism and hormone biosynthesis (Hayashi and Nishimura 2006; Hu et al. 2012; Reumann and Bartel 2016; Cross et al. 2016; Kao et al. 2018; Ronghui et al. 2020). In contrast to animals, in which fatty acid beta-oxidation occurs in both peroxisomes and mitochondria, plants carry out this pathway exclusively in peroxisomes, similar to yeast (see Sect. “Visualisation of peroxisomes in yeast cells”). Hence, plant peroxisomes are indispensable during early development, when seedlings depend on lipid breakdown prior to photosynthesis initiation (Graham 2008). Additionally, peroxisomes are integral to the photorespiratory pathway, functioning with chloroplasts and mitochondria to recycle phosphoglycolate, a toxic by-product of the oxygenase activity of Rubisco, thereby preventing carbon loss and maintaining photosynthetic efficiency under high light and low CO_2_ conditions (Dellero et al. 2016; Timm and Hagemann 2020). A common vital function of peroxisomes shared by plants is the detoxification of ROS, particularly hydrogen peroxide (H_2_O_2_), which is generated as a by-product of various oxidative reactions (Corpas et al. 2020). In this regard, studies in plants have been instrumental in advancing our understanding of the role of peroxisomes in cellular redox homeostasis and signalling (Corpas et al. 2019; Sandalio et al. 2023). Beyond these core functions, peroxisomes are also involved in the biosynthesis of important plant hormones such as jasmonic acid, which regulates defence responses against herbivores and pathogens, and the conversion of indole-3-butyric acid (IBA) to active auxin, a critical growth regulator (Delker et al. 2007). The physiological importance of peroxisomes extends to their role in plant responses to environmental stresses. These organelles are highly responsive to changes in cellular redox status (Lopez-Huertas et al. 2000), nutrient availability, and abiotic stress conditions such as high light, drought, salinity and heavy metal toxicity. Under oxidative stress, peroxisomes proliferate and enhance their ROS-scavenging capacity, thereby contributing to cellular homeostasis (Hinojosa et al. 2019). Furthermore, peroxisomes interact closely with other organelles, including chloroplasts, mitochondria and the ER, forming metabolic networks that optimize resource allocation and stress adaptation (Sparkes 2018; Liu and Li 2019; Oikawa et al. 2019; Koenig et al. 2023; Hall et al. 2024). Another important research area at the crossroads of fungal and plant research is the role of peroxisomes in the fungal infection of plants (Falter and Reumann 2022).

Electron microscopy and biochemical characterisations have revealed the diversity of peroxisomes in plant cells. Early ultrastructural studies of plant fine structures showed that plant “microbodies” are a class of single membrane-bound organelles, with a granular matrix containing crystalline, fibrous or amorphous inclusions, exhibiting ultrastructural similarities to animal peroxisomes (microbodies) (Frederick et al. 1968; Frederick and Newcomb 1969b). Biochemical studies have demonstrated that these organelles, containing catalase and α-hydroxyacid oxidases, can be isolated from various plant organs, each serving specialised functions (Tolbert et al. 1969). Notably, peroxisomes isolated from green leaves were shown to contain glycolate oxidase, glyoxylate reductase, and catalase, which are crucial for processes such as photorespiration and ROS detoxification (Tolbert et al. 1968). In parallel, a distinct class of specialised peroxisomes known as glyoxysomes was identified in the endosperm of castor bean (*Ricinus communis*) seedlings (Breidenbach and Beevers 1967; Breidenbach et al. 1968). These organelles are enriched in enzymes of the glyoxylate cycle, such as isocitrate lyase and malate synthase, as well as fatty acid beta-oxidation machinery and catalase, allowing for the conversion of lipids into carbohydrates during seed germination (Cooper and Beevers 1969). Phylogenetic analyses demonstrated that these glyoxylate cycle enzymes evolved from ancestral TCA cycle components and have been conserved in plants, fungi and bacteria, but lost in most metazoans (Kondrashov et al. 2006). Such evolutionary and functional diversity has enabled plants to develop specialised peroxisome types with distinct tissue-specific and developmental deployment, while maintaining the characteristic core metabolic functions of peroxisomes.

### Visualising plant peroxisomes by DAB cytochemistry: insights into plant peroxisome transitions

Following its successful use in mammalian studies (see Sect. “Visualising mammalian peroxisomes by DAB cytochemistry for peroxisomal catalase: the development of a peroxisome-specific staining method”), DAB staining was adapted to plant peroxisome research, enabling peroxisome visualisation in diverse physiological contexts such as photorespiration, developmental transitions and stress responses. The technique’s foundational validation in plants was achieved through complementary studies by Frederick and Newcomb (1969b), who employed DAB cytochemistry to localise catalase activity in spinach leaf microbodies (peroxisomes), and Vigil (1969), who demonstrated its application in glyoxysome-rich castor bean (*Ricinus communis*) endosperm. Importantly, inhibition experiments with 3-amino-1,2,4-triazole (a catalase-specific inhibitor) confirmed the staining’s dependence on catalase activity, establishing DAB as a specific marker for plant peroxisomes (Frederick and Newcomb 1969a). Subsequent studies further employed DAB cytochemistry to map peroxisome dynamics in photosynthetic tissues and germinating seeds—including the glyoxysome–peroxisome transition. For instance, in photosynthetic tissues, DAB-positive signals were closely associated with chloroplasts, reflecting the spatial coordination between peroxisomes and chloroplasts during glycolate metabolism. The utility of DAB staining extended to elucidating metabolic reprogramming during post-germinative growth, particularly in oilseeds where peroxisomes facilitate the conversion of stored lipids into carbohydrates. In castor bean endosperm, cytochemical studies using DAB demonstrated that glyoxysomes proliferated during early germination to mobilize lipid reserves but later transformed into peroxisomes as photosynthetic activity commenced (Vigil 1970). In addition, DAB staining has proven instrumental in revealing peroxisome behaviour under oxidative stress, where organelle proliferation often serves as a metabolic hallmark. Ultrastructural evidence from *Pisum sativum* systems revealed two paradigmatic cases: (1) chemical stress triggered by clofibrate, which stimulated a fivefold increase in peroxisome numbers (Palma et al. 1991) and (2) age-dependent stress during senescence, where peroxisomal populations expanded alongside escalating activated oxygen species production (Pastori et al. 1994).

### Visualising glyoxysomes by ferricyanide cytochemistry

While DAB staining is a robust tool for detecting catalase activity in peroxisomes, it cannot distinguish glyoxysomes from other peroxisomes. To address this limitation, researchers employed ferricyanide-based cytochemistry, which targets malate synthase, a key glyoxylate cycle enzyme exclusive to glyoxysomes. In this reaction, malate synthase releases free CoA, reducing ferricyanide to ferrocyanide (Hatchett’s brown). The resulting electron-dense deposits localise uniformly within the glyoxysome matrix, though minor nonspecific cytoplasmic staining may occur as a result of hydrolase activity or endogenous substrate interference (Trelease et al. 1974; Vaughn 1985). This method proved particularly valuable in fat-storing seedlings, where glyoxysomes dominate during early germination to facilitate lipid-to-carbohydrate conversion. Notably, dual staining approaches, combining ferricyanide for glyoxysomes with DAB for peroxisomes, revealed a metabolic transition: in cucumber cotyledons, glyoxysomes and peroxisomes were shown to coexist within the same microbody population as germinating seedlings shifted from lipid metabolism, mediated by the glyoxylate cycle, to photorespiration (Burke and Trelease 1975).

### Tracing reactive oxygen: how the cerium chloride technique illuminated plant peroxisomes

Like for peroxisomes from opisthokonta (see Sects. “Mapping oxidative activity: visualising peroxisomal oxidase activity in mammals by the cerium technique” and “Cerium cytochemistry and yeast peroxisomes: towards an understanding of peroxisome architecture”), the CeCl_3_ technique has been successfully employed for the cytochemical localisation of distinct oxidases in plant systems. Early studies applying this method successfully localised glycolate oxidase activity in leaf peroxisomes, glyoxysomes and unspecialised peroxisomes of higher plant tissues, achieving high specificity with minimal background staining (Thomas et al. 1981). However, further investigations revealed significant heterogeneity in enzyme distribution, with both reactive and non-reactive peroxisomes observed even after prolonged incubation (up to 36 h) (Kausch et al. 1983). These findings suggested that variations in glycolate oxidase content among peroxisomes may reflect differences in their physiological states within individual cells.

The CeCl_3_ technique was also employed for the cytochemical localisation of urate oxidase in the microbodies of root nodules by capturing peroxide generated during enzymatic urate oxidation. Cerium perhydroxide reaction deposits were exclusively observed in microbodies (peroxisomes), confirming biochemical fractionation data on urate oxidase distribution (Vaughn et al. 1982). Moreover, the CeCl_3_ method successfully detected urate oxidase in the small peroxisomes of rhizobia-infected cells, whereas the indirect DAB method failed to produce a clear positive reaction in these organelles. This demonstrated that the CeCl_3_ technique exhibited significantly higher sensitivity in revealing urate oxidase activity in such cases (Kaneko et al. 1987).

### Visualising plant peroxisomes by immunolabelling techniques: towards a characterisation of glyoxysomes

Immunocytochemical visualisation by electron microscopy is a straightforward method for the localisation of peroxisomal enzymes in animal tissues (see Sect. “Visualisation of mammalian peroxisomes by immunolabelling techniques: defining organelle identity and function”), but its application in plant systems presents unique technical challenges due to fundamental structural and biochemical differences between plant and animal cells. A primary obstacle is the presence of the plant cell wall, which acts as a diffusion barrier for macromolecules such as antibodies. Nevertheless, immunocytochemical techniques have been successfully adapted for plant studies by incorporating enzymatic digestion of the cell wall prior to antibody labelling (Wick et al. 1981). Plants contain endogenous substances such as lectins and glycoconjugates that non-specifically bind to immunoglobulins, leading to false-positive signals. These interference effects can be effectively eliminated by sequential blocking with pre-immune serum followed by unconjugated protein A before application of gold-conjugated detection reagents (Sautter 1984).

Immunocytochemistry on cryosections of lipid-rich, fat-storing tissues (e.g. in seedlings) presents particular challenges. Warming the sections can cause unfixed lipids to spread across the surface, obscuring antigenic sites and compromising ultrastructural integrity. Conversely, heat-sensitive enzymes are not compatible with conventional epoxy resin embedding, which requires high-temperature polymerization. To address these limitations, the Lowicryl technique, combined with protein A–gold labelling, has been successfully used to localise glyoxysomal enzymes in cotyledons (Sautter 1985).

Optimized conditions for immunogold labelling were evaluated to determine how well antigenicity and ultrastructure are preserved in plant tissues. Antigenicity was best maintained in LR White acrylic resin, whereas Spurr’s epoxy resin provided superior ultrastructural preservation (Van den Bosch and Newcomb 1986). Lowicryl resin, which is polymerized under ultraviolet light at low temperature, avoids the denaturing effects associated with heat and chemical polymerization in LR White. However, plant tissues embedded in Lowicryl showed poorer structural preservation than those embedded in LR White (Vaughn 1989).

**Fig. 5:**
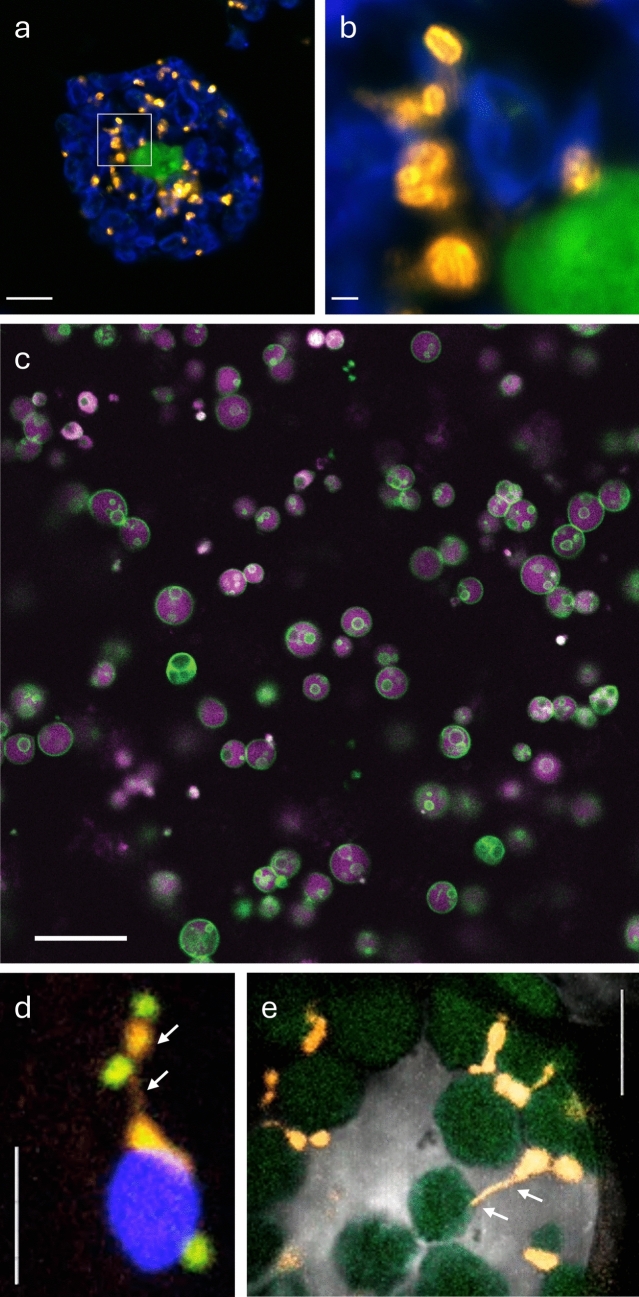
**a**, **b** Protoplasts isolated from 4-week-old *Arabidopsis thaliana* were immunostained with a PEX14-specific primary antibody and visualised by confocal microscopy using an Alexa Fluor 488-labelled secondary antibody (orange). Chlorophyll autofluorescence is shown in blue, and nuclei were stained with DAPI (green). Scale bar 10 µm; **b** zoomed-in region: 1 µm (kindly provided by C.-C. Lin and A. Baker, University of Leeds, UK). **c** Confocal micrograph of cotyledon cells of a 3-day-old wild-type *A. thaliana* seedling expressing a reporter that marks peroxisome membranes (mNeonGreen-mPTS) (green) and the peroxisome lumen (mRuby3-PTS1) (magenta). Scale bar 20 µm (kindly provided by N. Tharp and B. Bartel, Rice University, Houston, TX, USA). **d** Peroxisome protrusions (peroxules) in a transgenic *Arabidopsis* line expressing reporters that label peroxisomes (YFP-PTS1) and mitochondria (mitoGFP). Time-lapse snapshot of a hypocotyl cell showing a chloroplast (blue) with an associated peroxisome (orange) extending a peroxule (arrows) contacting mitochondria (green). Scale bar 5 μm (see Jaipargas et al. 2016 for further information). **e** Interaction of peroxisomes/extended peroxules with chloroplasts in a transgenic *Arabidopsis* line. Combination of bright field and fluorescence imaging. The chloroplast autofluorescence has been false-coloured green. The peroxisomes/extended peroxules are labelled with YFP-PTS1. Scale bar 5 μm (**d**, **e** kindly provided by J. Mathur, University of Guelph, ON, Canada)

Immunogold labelling has been extensively utilized to investigate the subcellular localisation and dynamic transformations of peroxisomes and glyoxysomes in plants by targeting their characteristic marker enzymes. Early studies demonstrated the efficacy of this approach through the specific labelling of glyoxysomes in cottonseed using antibodies against the glyoxylate cycle enzyme isocitrate lyase (ICL). This is the first report of an ultrastructural localisation of a glyoxysomal enzyme by post-embedding immunocytochemistry (Doman et al. 1985). Subsequent double-label immunoelectron microscopy targeting both peroxisomal and glyoxysomal enzymes revealed that glyoxysomes transform into peroxisomes during cotyledon germination, while peroxisomes revert to glyoxysomes during leaf senescence (Titus and Becker 1985; Nishimura et al. 1986, 1993; Sautter 1989). Immunogold labelling has also been instrumental in characterising peroxisomal matrix organisation, including the localisation of catalase in both amorphous and crystalline inclusions (Tenberge and Eising 1995; Tenberge et al. 1997). In addition, the technique has also proven valuable for studying specialised peroxisome functions in different plant tissues, such as nodule-specific urate oxidase (uricase), which was used as a marker enzyme to study its distribution during nodule development (Vaughn and Stegink 1987; Van den Bosch and Newcomb 1986). Immunogold labelling was also employed to localise a new peroxisomal channel protein (PCP) in bromegrass. This protein was found to be induced by abscisic acid (ABA), cold and drought stresses, as well as during late embryogenesis (Wu et al. 2005). Moreover, Banjoko and Trelease (1995) used immunofluorescence to monitor protein import into peroxisomes, and revealed that the C-terminal SKL tripeptide, a consensus-targeting signal for mammalian peroxisomes, also targets proteins to plant peroxisomes. Recently, immunofluorescence and confocal microscopy were applied to show that the in vivo trafficking of plant CAT2 to peroxisomes and the nuclei is redox-regulated (Lin et al. 2025) (Fig. 5).

As in mammalian and yeast systems (see Sects. “Visualisation of mammalian peroxisomes by immunolabelling techniques: defining organelle identity and function” and “Immunolabelling of yeast peroxisomes: implications for protein import and function”), epitope tagging has become a key approach for investigating the subcellular localisation of peroxisomal proteins in plant cells. By fusing peroxisomal membrane proteins to small, well-characterised epitope tags, researchers can take advantage of commercially available antibodies to detect these fusion proteins with high specificity. For example, Murphy et al. (2003) successfully localised the peroxisomal membrane protein PMP22 in *Arabidopsis thaliana* by generating transgenic lines expressing MYC-tagged PMP22 and visualising its distribution using anti-MYC immunodetection. Similarly, the localisation of *Arabidopsis* PEX16, a peroxin essential for peroxisome biogenesis, has been characterised using a comparable strategy (Karnik and Trelease 2005).

### Fluorescent fusion proteins and bioreporters in plant peroxisome research: new insights into peroxisome dynamics and physicochemical properties

Green fluorescent protein (GFP) has been extensively applied as a versatile molecular tool to study the structure, dynamics and interactions of peroxisomes in living plant cells (see also Sects. “Visualisation of mammalian peroxisomes by expression of fluorescent fusion proteins with a peroxisomal targeting signal: from static images to dynamic organelles” and “Visualising yeast peroxisomes by fluorescent fusion proteins: insights into peroxisome dynamics, inheritance, de novo formation and organelle contacts”). By fusing GFP (or variants) to peroxisomal targeting signals such as PTS1 or PTS2, researchers have visualised plant peroxisomes in real time across different tissues and developmental stages (Goto-Yamada et al. 2015) (Fig. 5). GFP labelling enabled detailed observation of peroxisomal morphology in plants, ranging from spherical to tubular forms, and tracks various types of motilities, including oscillatory and directional movements (Mano et al. 2002; Jedd and Chua 2002). Furthermore, GFP-based imaging has uncovered peroxisome accumulation at the cell division plane during mitosis and cytokinesis in onion and leek cells. This redistribution was shown to be actomyosin-dependent and likely contributes to membrane recycling or redox regulation during cell plate formation (Collings et al. 2003). Beyond localisation, GFP-tagged mTalin (mouse talin actin-binding domain) along with yellow fluorescent protein (YFP) fused to a peroxisomal targeting signal containing the SKL motif has demonstrated that peroxisomes associate predominantly with actin filaments rather than microtubules (Mathur et al. 2002) Similarly, GFP-tagged peroxisomal proteins such as multifunctional protein (MFP) have revealed unexpected dual localisations and interactions with cortical microtubules, suggesting broader regulatory functions beyond peroxisomal metabolism (Chuong et al. 2005). Further dissection of cytoskeletal dependencies using GFP-based imaging has confirmed that peroxisomal movement and positioning are largely actin-dependent, with microtubules playing only a minor role (Collings et al. 2011). An intriguing aspect of plant peroxisome dynamics is the formation of long membrane extensions called peroxules, which have been associated with ROS stress and organelle interaction in plant cells (Rodriguez-Serrano et al. 2016; Jaipargas et al. 2016; Mathur 2021) (Fig. 5). Interestingly, similar tubular extensions can also be observed in mammalian cells (reviewed in Carmichael et al. 2023) (see Sect. “Visualisation of mammalian peroxisomes by expression of fluorescent fusion proteins with a peroxisomal targeting signal: from static images to dynamic organelles”).

In addition to structural imaging, functional fluorescent probes have expanded the scope of peroxisomal research in plants. For example, Cameleon, a peroxisome-targeted calcium sensor composed of two modified GFPs (donor and acceptor) linked via calmodulin and a calmodulin-binding peptide, enabled real-time monitoring of peroxisomal calcium levels. Upon Ca^2+^ binding, the interaction between the two GFPs increases FRET efficiency, resulting in changes in the donor/acceptor fluorescence ratio that reflect intracellular Ca^2+^ concentrations (Chaerle and Straeten 2001). Bimolecular fluorescence complementation (BiFC) has also been employed to investigate protein–protein interactions at peroxisomes, such as the complex formation between PEX7 and PEX12 in living plant cells (Singh et al. 2009). Moreover, oxidative stress-related fluorescent probes (e.g. DCF-DA, 2′,7′-dichlorofluorescin diacetate) or reporters have been used to detect intracellular hydrogen peroxide (H_2_O_2_) or ROS, offering further insight into peroxisomal roles in cellular redox balance (Samuilov et al. 2008; Lim et al. 2019; Arnaud et al. 2023).

Photoconvertible fluorescent proteins, such as Kaede, are powerful tools for tracking the dynamic behaviour of organelles such as peroxisomes in living cells. These proteins can change their fluorescence emission properties upon exposure to specific wavelengths of light. Kaede undergoes a permanent shift in fluorescence emission (e.g. from green to red) when exposed to violet or blue light (typically around 405 nm). By targeting these proteins to peroxisomes (e.g. by fusing them with a peroxisomal targeting signal, PTS1 or PTS2), researchers could “mark” a subpopulation of plant peroxisomes at a given time and track their movement, interactions, fusion, division or degradation over time using time-lapse confocal or spinning-disk microscopy (Fujimoto et al. 2009).

While GFP is widely utilized for visualising protein localisation in live cell imaging by fusing it to a protein of interest, the relatively large size of even the smallest fluorescent protein variants can interfere with proper protein folding, function or subcellular targeting. Jean-Denis Pédelacq first introduced superfolder GFP (sfGFP), a GFP variant engineered through mutagenesis to exhibit enhanced folding efficiency, structural stability and rapid attainment of its native conformation (Pedelacq et al. 2006). A notable application of sfGFP technology in plant peroxisome research was demonstrated by Al-Hajaya et al., who developed a two-component system consisting of sfGFP1-10 fused to catalase and a 13-amino acid sfGFP11 peptide to investigate peroxisomal protein import in plants (Al-Hajaya et al. 2023). A wide variety of fluorescent proteins (FPs) are now available, featuring diverse physicochemical properties such as emission spectra, brightness, quantum yield, maturation rate, p*K*_a_ and fluorescence lifetime, which are continuously refined and expanded by the research community (https://www.fpbase.org/). For instance, the fluorescent protein mRuby3, when fused to a peroxisomal targeting signal (PTS), enables visualisation of the peroxisomal matrix. In parallel, mNeonGreen tagged with a membrane-specific PTS (mPTS) selectively labels the peroxisomal membrane. This dual-labelling strategy has uncovered the presence of complex internal membrane structures within peroxisomes in *Arabidopsis* (Wright and Bartel 2020; Tharp et al. 2026) (Fig. 5). Beyond fluorescent proteins, small-molecule probes such as BODIPY dyes have been applied to live imaging of peroxisomes in plant cells (Landrum et al. 2010). In *Arabidopsis*, Nitro-BODIPY has been used to monitor peroxisome proliferation under salt stress, detect peroxisome aggregation during cell death, and reveal peroxisome accumulation in autophagy-deficient mutants (Fahy et al. 2017).

### Advancing plant peroxisome research through super-resolution microscopy

Super-resolution microscopy in plant peroxisome research is still in its early stages, but emerging techniques such as ROOT-ExM, ClearSee and SIM-compatible labelling strategies are opening exciting new avenues. Plant root expansion microscopy (ROOT-ExM) enables nanoscale imaging (approx. 70 nm resolution) by physically expanding plant tissues. It has been used to visualise various organelles and holds strong potential for future peroxisome imaging, especially when combined with immunolabeling and light-sheet microscopy (Grison et al. 2024). However, expansion microscopy for peroxisomes appears to be challenging according to studies in mammalian (Buttner et al. 2021) and yeast cells (E. Zalckvar, personal communication). Although tissue clearing methods such as ClearSee, which reduce autofluorescence and enhance deep-tissue imaging, and structured illumination microscopy (SIM), which doubles resolution compared to conventional fluorescence microscopy, have not yet been directly applied to peroxisome imaging in plants, their demonstrated success in other organelles suggests strong future potential. Together, these technologies are expected to form a powerful toolkit for studying the ultrastructure and dynamics of peroxisomes and other organelles in complex plant tissues.

## Conclusions

Peroxisomes have emerged as central metabolic and signalling organelles whose roles in lipid metabolism and redox homeostasis underpin a wide range of physiological and pathological processes. The recognition of their involvement in antiviral responses, innate immunity, cancer, and neurodegenerative and age-related disorders underscores their broad medical relevance. As outlined in this review, advances in peroxisome biology have been tightly linked to the development of morphological techniques that enabled their specific identification and visualisation across mammals, yeast and plants. From early cytochemical staining and electron microscopy to contemporary fluorescence-based and high-resolution imaging approaches, each methodological innovation has provided new insights into peroxisome structure, dynamics and function. Placing these techniques in a historical and methodological context highlights how imaging has shaped our current understanding of peroxisomes. To date, cytochemical staining methods such as DAB cytochemistry are still valuable tools to identify peroxisomes, in particular in tissues and organisms which are not well characterised, challenging with respect to transfection and expression of peroxisomal marker proteins, and where specific antibodies are lacking. Cryo-electron microscopy is currently revealing novel and exciting insights into the structure of peroxisomal membrane protein complexes in yeast and mammals (e.g. Blok et al. 2015; Vonck et al. 2016; Feng et al. 2022; Rüttermann et al. 2023; Bürgi et al. 2023; Li et al. 2024). Moreover, advances in super-resolution microscopy will result in more detailed insights into the peroxisomal matrix and membrane organisation and function, as will the increasing toolbox of peroxisomal bioreporters. Furthermore, the rapid development of AI-based image analysis and machine learning tools will improve screening approaches and the quantification of dynamic peroxisome alterations and interactions. The visualisation and imaging of peroxisomes will continue to be essential for addressing outstanding questions regarding their biogenesis, regulation, heterogeneity, organelle communication and roles in health and disease.

## Data Availability

No datasets were generated or analysed during the current study.
